# Death of Dr. Kennedy

**Published:** 1892-07

**Authors:** 


### DEATH OF DR. L. P. KENNEDY.

Dr. L. P. Kennedy, who had been quite ill for several months with typhoid fever, died at his former home in Due West, S C., early in June. Dr. Kennedy was a graduate of Erskine College, South Carolina. He then spent one year at the Johns Hopkins University. He received his medical education from the Medical Department of the University of the City of New York, class of 1887.

After graduating, he accepted a position in the hospitals on Blackwell's Island for six months, and then spent nine months in Charity Hospital, Brooklyn.

Of him, Rev. W. M. Greer, D. D., President of Erskine College, once wrote: "He is a pleasant speaker, has a clear, musical voice, and while not an orator, has the capacity for strong, clear-cut, compact speech."

As assistant to the chair of obstetrics and diseases of women in the Atlanta Medical College, as one of the editors and proprietors of the *Atlanta Medical and Surgical Journal* and as a practitioner, he took the highest rank.

One of the editors of the RECORD, Dr. J. McFadden Gaston, has been absent during the month of June, first at Detroit in attendance upon the American Medical Association, and then at Philadelphia, actively studying the new methods of surgery. Dr. Gaston has been royally entertained at the homes of Drs. John H. Packard, John Ashhurst, Jr., J. H. Musser, and others, and has also been interested in the operations of these distinguished surgeons, as well as those of Drs. E. E. Montgomery, Joseph and Mordecai Price, Ernest Laplace, and Wharton. Dr. J. B. Roberts has had some very important cases to show Dr. Gaston, one of which was the removal of the spinous processes and arches of the seventh, eighth, and ninth dorsal vertebræ. We hope to have a letter from Dr. Gaston about his recent stay in the City of Brotherly Love.

